# Effect of focused ultrasound-induced mechanical ablation on stemness and dormancy properties of residual/peri-focally localized glioblastoma cells

**DOI:** 10.1093/noajnl/vdaf184

**Published:** 2025-08-30

**Authors:** Dana Hellmold, Levi Johanning, Jacqueline Clüver, Jonna Holler, Nils Oliver Schröder, Frieda Bayler, Hajrullah Ahmeti, Carolin Kubelt-Kwamin, Sina Wieker, Ann-Kristin Helmers, Michael Synowitz, Janka Held-Feindt

**Affiliations:** Department of Neurosurgery, University Medical Center Schleswig-Holstein (UKSH), 24105 Kiel, Germany; Department of Neurosurgery, University Medical Center Schleswig-Holstein (UKSH), 24105 Kiel, Germany; Department of Neurosurgery, University Medical Center Schleswig-Holstein (UKSH), 24105 Kiel, Germany; Department of Neurosurgery, University Medical Center Schleswig-Holstein (UKSH), 24105 Kiel, Germany; Department of Neurosurgery, University Medical Center Schleswig-Holstein (UKSH), 24105 Kiel, Germany; Department of Neurosurgery, University Medical Center Schleswig-Holstein (UKSH), 24105 Kiel, Germany; Department of Neurosurgery, University Medical Center Schleswig-Holstein (UKSH), 24105 Kiel, Germany; Department of Neurosurgery, University Medical Center Schleswig-Holstein (UKSH), 24105 Kiel, Germany; Department of Neurosurgery, University Medical Center Schleswig-Holstein (UKSH), 24105 Kiel, Germany; Department of Neurosurgery, University Medical Center Schleswig-Holstein (UKSH), 24105 Kiel, Germany; Department of Neurosurgery, University Medical Center Schleswig-Holstein (UKSH), 24105 Kiel, Germany; Department of Neurosurgery, University Medical Center Schleswig-Holstein (UKSH), 24105 Kiel, Germany

**Keywords:** mechanical focused ultrasound, glioblastoma, dormancy, stemness

## Abstract

**Background:**

Focused ultrasound (FUS) is a new technology that enables the spatially and temporally precise delivery of ultrasound energy to various targets. In addition to its known applications in treating tumors, cavitation-based mechanical focused ultrasound (mFUS) is gaining importance. Due to the novelty of this technique, little is known about the effects of mFUS on peri-focally localized or surviving tumor cells. Glioblastomas (GBMs) are highly malignant intracranial tumors with a pronounced intra- and intertumoral heterogeneity, which, eg leads to their evasion of appropriate treatment regimens.

**Methods:**

The impact of mFUS was investigated in patient-derived GBM organoids (GBOs), glioma stem-like cells (GSCs), and differentiated GBM cells in an in vitro 3D hydrogel culture model. Particular attention was paid to investigating the stemness and dormancy properties of residual/peri-focally localized GBM cells, as these may be important for tumor progression.

**Results:**

In GBOs and different primary cells, increased expression of dormancy- and stemness-associated markers was found in a complex region- and marker-dependent manner mediated via PI3-kinase/Akt/GSK3β signaling, suggesting an effect of mFUS beyond the focal area. mFUS resulted in an increased ability of residual/peri-focal, formerly differentiated patient-derived GBM cells to form stem cell-typical spheres associated with increased expression of various dormancy and stemness markers. Residual/peri-focal patient-derived cells were characterized by a higher resistance to temozolomide, resulting in fewer dead cells compared to temozolomide treatment alone.

**Conclusion:**

The ablation of defined regions by mFUS appears to regulate the stemness and dormancy properties of the residual/peri-focally localized GBM cells in a region-specific manner.

**Key Points**

- Mechanical FUS is a relevant future therapy for treating brain tumors.
- Presentation of an in vitro mechanical FUS organoid/3D cell model.
- Mechanical FUS regulates dormancy/stemness properties of residual GBM cells.

**Importance of the Study**

Ablation with mechanically focused ultrasound (mFUS) is considered an important future therapy for the treatment of malignant brain tumors. This study is the first to demonstrate the effects of mFUS on the dormancy and stemness properties of surviving or peri-focally localized glioblastoma (GBM) cells. Since commercially available in vivo mFUS systems for mechanical ablation of brain tumors in rodents are limited, the studies were performed in an in vitro mFUS setup using patient-derived GBM organoids, glioma stem-like cells (GSCs), and differentiated GBM cells. Dormancy- and stemness-associated molecules were regulated by mFUS in a complex region- and marker-dependent manner, and the residual/peri-focal GBM cells exhibited enhanced characteristics of GSCs. As mFUS represents an important future therapeutic option and dormancy and stemness properties may play a central role in tumor progression, the results may provide a basis for a better understanding of the clinically relevant effects of mFUS in GBMs.

Focused ultrasound (FUS) is a new technology that enables spatially and temporally precise delivery of ultrasound energy. Various FUS effects using continuous (cFUS) or pulsed ultrasound (pFUS) are used for therapeutic purposes. Here, cFUS provides a localized increase in temperature above 43 °C to induce permanent tissue necrosis or thermal lesions in the focal area of the transducers.^1,2^ In comparison, pFUS mediates a nonthermal mechanism, the so-called cavitation effect.^3^ The cavitation effect describes the formation, growth, and collapse of gas-filled bubbles in liquids under the influence of an ultrasonic field.^3^ In the so-called stable cavitation at low sound intensity, the bubbles exist over several cycles, oscillate, and maintain a stable resonance size. In contrast, inertial cavitation at high sound pressure describes bubbles that exist for only a few cycles, as they regularly collapse.^3^ The oscillation and collapse of bubbles under the influence of pFUS lead to an abrupt release of energy and generation of oxygen species, shock waves, and microjets, disrupting the cell membrane and ablating the surrounding tissue structure.^3–6^ Therefore, this FUS mechanism is also called high-intensity mechanical FUS (mFUS). Since mFUS is characterized by less thermal energy deposition, which protects the surrounding healthy tissue,^7,8^ it is also becoming increasingly important in medical therapy. Recent research has shown that mFUS is a promising mechanism for treating malignant brain tumors and is a relevant future therapy.^4,5,7^

Glioblastomas (GBM) are highly malignant primary intracranial tumors characterized by rapid progression and poor patients’ prognosis due to frequent relapses and resistance to chemo- and radiotherapy.^9,10^ Indeed, subpopulations with stem cell characteristics, so-called glioma stem-like cells (GSCs), which are slowly cycling, have tumorigenic potential, are pluripotent and show high resistance to chemotherapy, as well as dormant tumor (single) cells, which reversible enter a resting state (cellular dormancy) to escape from (chemo)therapeutic treatment, are closely associated with the aggressiveness of GBMs.^11–15^ Dormant and tumor stem-like cells share some similarities,^16,17^ can wax and wane depending on environmental conditions, may increase the aggressive potential of GBMs, and may trigger GBM recurrence.^11–13,18^

Despite these challenges, recent studies have demonstrated successful transmission of ultrasound waves for thermoablation of GBMs.^19,20^ In addition, the opening of the blood–brain barrier by low-intensity FUS or the activation of chemical compounds known as sonosensitizers, which lead to cell death (sonodynamic therapy), has been extensively studied.^7,19,21^ Regarding the potential use of mFUS in GBM, Sukovich et al. have shown in a first in vivo study that a homemade mechanical ablation device can produce well-defined lesions in the porcine cerebral cortex after partial craniectomy.^22^ Furthermore, a team of researchers at the University of Michigan recently developed a transcranial MR-guided mFUS system. It successfully created target lesions in 2 ex vivo porcine brains encased in a human skull without causing major hemorrhage or edema. Moreover, they started to evaluate the transcranial mFUS parameters required to study mouse brain tumor models.^23–25^ However, due to significant attenuation and deviations in the passage of ultrasound through the skull,^5^ current commercially available in vivo mFUS systems can only be used for mechanical ablation of brain tumors with reservations and require further development.

Accordingly, given the pronounced cell heterogeneity and high cellular adaptability of GBMs, it is clear that further studies are urgently needed to better understand this technique’s biological impact. Thus, this study investigates the effects of mFUS on patient-derived glioblastoma organoids (GBOs) and primary GBM cells as a novel therapeutic approach in an in vitro 3D hydrogel culture model. Since the stemness and dormancy properties of GBM cells may play a central role in tumor progression,^11–15^ a particular focus was placed on investigating the effect of mFUS on these properties in peri-focally localized or surviving tumor cells.

## Materials and Methods

### Preparation of Patient-derived Glioblastoma Organoids/Tumoroids

Patient-derived glioblastoma organoids/tumoroids (GBOs) were prepared according to the protocol published by Jacob et al.^26^ Human GBM tissue samples were obtained directly from surgical resections performed by the Department of Neurosurgery (University Medical Center Schleswig-Holstein, Kiel, Germany), with the approval of the Ethics Committee of the University of Kiel, Germany, after the written informed consent of donors (file reference: D524/17) and following the Helsinki Declaration of 1975. According to World Health Organization (WHO) criteria, the tissue samples were diagnosed and classified by a neuropathologist as IDH wild-type GBMs, CNS WHO grade 4 (University Medical Center Hamburg-Eppendorf, UKE, Hamburg, Germany).

**Figure 1.**
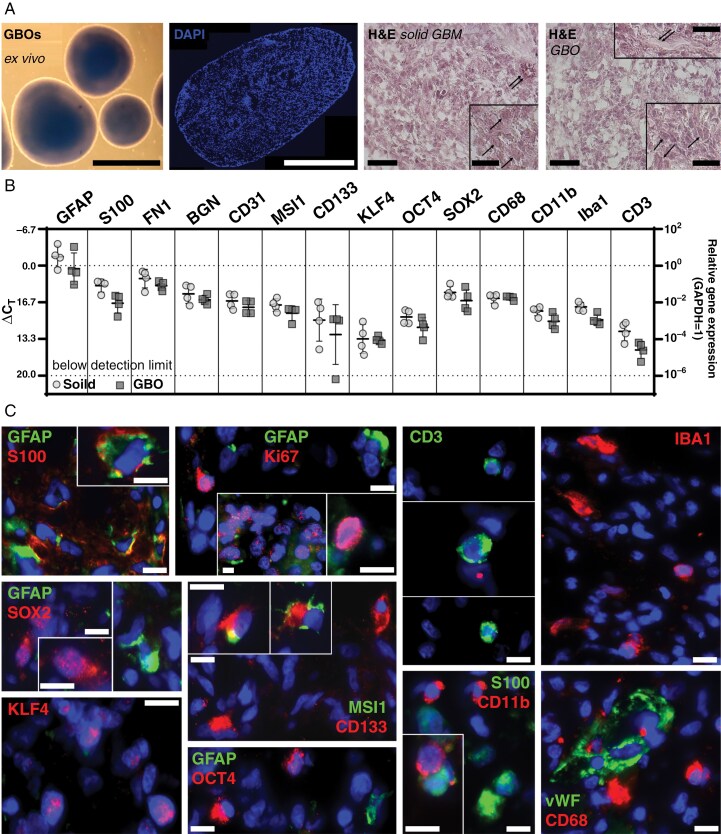
GBOs replicate the intra- and intertumoral heterogeneity in histological characteristics, cellular variety, and gene expression patterns of the freshly original tumors. (A) GBOs grown as vital cultures in vitro for 2 weeks (left), and after preparation of histological sections, followed by nuclear staining with DAPI (middle) or H&E staining compared to the corresponding solid parental tumor (right; single arrows point to individual tumor cells, double arrows point to vascular structures). (B) Comparison of the mRNA expression of various cell type-specific markers between *n*=4 independent GBO preparations and the corresponding parental tumors. BGN, biglycan; CD, cluster of differentiation. Significances between different stimulations were determined using an ordinary one-way ANOVA test followed by a Tukey’s multiple comparison test; no significances were detected due to the heterogeneity of the GBO preparations. (C) Immunofluorescence staining of GBOs with cell type-specific markers. DAPI, 4′,6-diamidino-2-phenylindole; FN1, fibronectin 1; GAPDH, glyceraldehyde-3-phosphate dehydrogenase; GFAP, glial fibrillary acidic protein; IBA1, calcium-binding adapter molecule 1; Ki67, Kiel67 (proliferation); KLF4, Krüppel-like factor 4; MSI1, Musashi (Drosophila) homolog 1; OCT4, octamer binding transcription factor 4; SOX2, sex-determining region Y-box 2; vWF, von-Willebrand-factor. Bar: 1 mm (A, left), 0.5 mm (A, middle); 100 µm (A right; inserts 20 µm); others (C) 20 µm.

The GBOs were cultured in ultra-low attachment 6-well plates (Corning, NY, USA) with GBO medium (consisting of 50% DMEM/F12 (Thermo Fisher Scientific, Waltham, MA, USA), 50% Neurobasal medium (Thermo Fisher Scientific), GlutaMAX™, nonessential amino acids (NEAAs; Thermo Fisher Scientific), penicillin–streptomycin, N2 and B27 supplements without vitamin A (Thermo Fisher Scientific), 2-mercaptoethanol (Thermo Fisher Scientific), and human insulin (Sigma–Aldrich, St. Louis, MO, USA; Merck Millipore)) without growth factors or fetal calf/horse serum as described.^26^ The plates were incubated on an orbital shaker (#8012-1771, Binder GmbH, Tuttlingen, Germany) at 120 rpm in a humidified incubator at 37 °C and 5% CO₂. The GBOs replicated the intra- and intertumoral heterogeneity of GBMs in terms of histological characteristics, cellular variety, and gene expression patterns of the original tumors (Figure 1A-C).

### Cultivation of Patient-derived Primary Glioblastoma Cells

Patient-derived primary GBM cells and GSCs were obtained by dissociating tumor material collected during surgical procedures performed at the Department of Neurosurgery, University Medical Center Schleswig-Holstein, Kiel, Germany. The process was approved by the University of Kiel’s ethics committee (file reference: D524/17), with written informed consent from donors, and adhered to the 1975 Declaration of Helsinki principles. The isolated GBM cells were cultured in DMEM (Thermo Fisher Scientific) supplemented with 10% (v/v) fetal bovine serum (FBS, Thermo Fisher Scientific) following previously established protocols.^27^ GSCs were maintained under stem-like conditions in F12 media supplemented with B27 supplement (Thermo Fisher Scientific), 2 mM L-glutamine, and 1% penicillin–streptomycin (10,000 U/mL). Epidermal growth factor (Peprotech, Cranbury, NJ, USA) and basic fibroblast growth factor (Immunotools, Friesoythe, Germany) were added at a concentration of 10 ng/mL as described before.^27^ GSCs were identified by their ability to form neurospheres, survive and proliferate under stem cell conditions, and differentiate into more mature cell types, as previously validated.^13,28,29^ The purity of different primary cells was confirmed through immunostaining with cell type-specific markers and by ensuring the absence of mycoplasma contamination.

### 3D Hydrogel GBO/Cell Cultures for In Vitro mFUS Application

For the 3D hydrogel cultures, VitroGel IKVAV-Hydrogel (The Well Bioscience, Monmouth Junction, NJ, USA) was used at a 1:5 dilution (stiffness corresponds to brain stiffness^30^) and was prepared according to the manufacturer’s protocol. To apply an in vitro mFUS treatment, the 3D hydrogel GBO/cell cultures were prepared directly in Covaris tubes (1 mL; Covaris LLC., Woburn, MA, USA) suitable for the in vitro mFUS setup. The hydrogel was prepared in Eppendorf tubes (Sarstedt; 1.5  mL; PP 72.690.300, Nürnbrecht, Germany) by mixing the dilution solution type I (The Well Bioscience) with IKVAV-hydrogel in a 1:5 dilution, which was then incubated for 10 min at room temperature (RT).

In the case of GBO 3D hydrogel cultures, approximately 30 GBOs were transferred into the Covaris tube, and any transferred medium was aspirated from the GBOs. The GBO medium was mixed in a ratio of 4:1 v/v with the hydrogel and then transferred onto the GBOs in the Covaris tubes. After 5 min at RT, the hydrogel began to polymerize, allowing the GBOs to be adjusted and positioned within the gel. The tubes were covered with Parafilm (Amcor, Zürich, Switzerland) and incubated at 37 °C with 5% CO₂ for 45 min. After polymerization, the hydrogel was carefully covered with GBO medium and cultured for another 24 h before mFUS application. The GBO medium was renewed directly before mFUS treatment.

In the case of 3D hydrogel cell cultures, the Covaris tubes and the IKVAV-hydrogel solution were prepared as described above. Depending on the cell type used, a cell suspension containing 24 × 10^6^ cells/ml in the respective growth media containing 5× critical growth factors was prepared. The cell suspension was added to the hydrogel solution in a 4:1 (v/v) ratio. The hydrogel–cell mixture was then transferred into the Covaris tubes. The tubes were covered with Parafilm (Amcor) and incubated at 37 °C with 5% CO_2_ for 45 min. After polymerization, the hydrogel was carefully covered with the respective growth medium containing 1× of the critical growth factors and cultured for another 24 h before mFUS application. The medium was renewed directly before mFUS treatment.

### In Vitro Mechanical Focused Ultrasound Setup

The M220 ultrasound generator with a 0.5-MHz solid-state ultrasound transducer and geometrically focused acoustic energy was used (Covaris LLC.; https://www.covaris.com/technology/afa-technology). The instrument was initially developed as a platform for fast and highly efficient FUS-induced mechanical preanalytical preparation of diverse biological samples. As defined by Covaris, the system generates a wavelength of only a few millimeters, which allows ultrasound energy to be focused in a focal zone within a sample vessel immersed in a water bath. Through local pressure fluctuations, focused ultrasonic energy bursts control the generation and collapse of millions of cavitation bubbles within the sealed sample vessel in an isothermal, noncontact environment that avoids thermal molecular damage. As defined by Covaris, in this process, the cavitation bubbles oscillate or grow to a critical size and then collapse, creating hydrodynamic shear stresses in the sample. The system allows (i) customization of the peak incident power (PIP, W), duty cycle (DF, %), cycles per burst (CPB, number), and duration (s) to match the AIP (W) of required mFUS settings while maintaining constant temperature conditions; and (ii) insertion of spherical reaction vessels of appropriate size (up to 1 ml).

**Figure 2.**
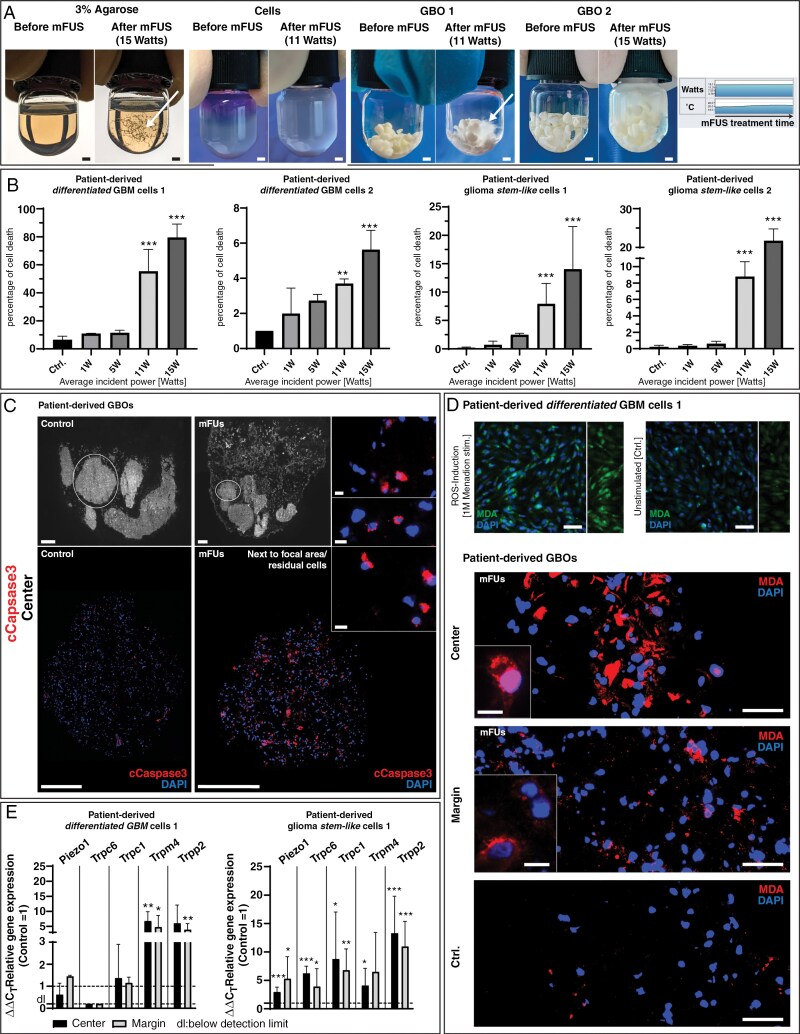
(A) Mechanical focused ultrasound (mFUS) resulted in visible agarose lesions and increased destruction in GBOs- or GBM cells-containing samples while temperature and wattage remained constant (white arrows point to destroyed agarose/GBOs in the focal area after mFUS). Bar: 1 mm. (B) mFUS settings with increasing average incident power resulted in increasing numbers of dead cells in pure cultures of patient-derived, more differentiated GBM cells and GSCs. The individual cultures reacted to varying degrees, so no consistently increased sensitivity of a specific cell population could be observed. *n* = 3 biological replicates of individual cell preparations with *n* = 1-2 technical replicates each; the significances between different stimulations were determined using an ordinary one-way ANOVA test followed by a Tukey’s multiple comparison test (** *P* < 0.01; *** *P* < 0.001). Error bars correspond to the standard deviation. (C) mFUS-treated (average incident power 11 W) GBO-containing samples cultured for 24 h after mFUS and cryofixed were serially sectioned. Immunofluorescence staining was performed with cCapase3 (cell death marker). cCaspase3 appeared predominantly in mFUS-treated samples compared to the untreated control. Bar: 0.4 mm (whole sections), 400 µm (GBO samples), 10 µm (inserts). Circled GBOs in whole sections show those GBOs that were visualized in detail in the immunofluorescence. (D) Determination of ROS after mFUS (average incident power 11 W) by MDA immunofluorescence staining. After validation of the specificity of MDA staining (induction of ROS without mFUS by 1-hour administration of Menadione (1 M) in pure cultures of more differentiated GBM cells), ROS generation after mFUS could be detected in GBO-containing samples to a significantly higher extent than in untreated control samples. Bar: 50 µM (overviews), 10 µm (inserts). (E) mRNA expression of ion channels (mechanoreceptors) following mFUS (average incident power 15 W) in pure cultures of patient-derived, more differentiated GBM cells and GSCs, focusing on piezo-type mechanosensitive ion channel component 1 (Piezo1), transient receptor potential canonical (Trpc) 1 and 6, transient receptor potential polycystic 2 (Trpp2), and transient receptor potential cation channel subfamily M member 4 (Trpm4). Increased expression of mechanoreceptors of all (GSCs) or Trpm4 and Trpp2 (more differentiated GBM cells) was detected after mFUS compared to individual, average unstimulated controls (control = 1); *n* = 1-2 biological replicates with *n* = 1-2 technical replicates each. Significant differences compared to the untreated control were determined by a nonpaired *t*-test and are indicated directly above the bars (* *P* < 0.05; ** *P* < 0.01; *** *P* < 0.001). Error bars correspond to the standard deviation.

The validation of the in vitro mFUS setup was performed by addressing several points: First, we showed that mFUS with high acoustic pressure under constant temperature conditions (average incident power [AIP] 1 W: PIP 10 W, DF 10%, CPB 100, 10 s; AIP 5 W: PIP 35 W, DF 15%, CPB 150, 10 s; AIP 11 W: PIP 55 W, DF 20%, CPB 200, 10 s; AIP 15 W: PIP 75 W, DF 20%, CPB 200, 10 s; pulse repetition frequency (PRF) of 300-350 Hz corresponded to preclinical studies using mechanical ablation for, eg liver tumors^4–6^) led to (macroscopically visible) defects in the focal area of exemplary agarose samples, as well as in GBM cells grown as a cellular network and GBOs, both embedded in hydrogels (Figure 2A). Next, when mFUS settings with increasing AIP were used, increasing numbers of dying GBM cells were detectable 24 h after mFUS (Figure 2B), regardless of whether pure cultures of patient-derived GSCs or more differentiated primary GBM cells were used. Here, the individual cultures reacted to varying degrees, so no consistently increased sensitivity of a specific cell population could be observed. In addition, pronounced expression of activated/cleaved (c)Caspase 3 as a cell death marker was observed in the residual cells, as illustrated for GBOs, predominantly near the focal region compared to more distant regions (AIP 11 W, 24 h after mFUS) (Figure 2C). Furthermore, since mFUS facilitates an abrupt release of energy, generating shear stress and reactive oxygen species (ROS),^3–6^ we examined whether we could detect ROS formation in our in vitro mFUS setup. Due to ROS’s short-lived nature, we indirectly examined ROS formation by detecting lipid oxidation resulting from the cells’ contact with ROS molecules through immunofluorescence staining of malondialdehyde (MDA; methodical details see below). MDA forms a degradation product of lipid hydroperoxides generated by the reaction of polyunsaturated fatty acids with ROS or free radicals.^31^ After validating the specificity of MDA staining (induction of ROS without mFUS by applying Menadione for 1 h in more differentiated GBM cells [1 M, dissolved in ethanol, #M57405, Sigma–Aldrich]), we demonstrated that ROS was generated in GBOs in our mFUS setup (AIP 11 W, 24 h after mFUS) (Figure 2D). Finally, we analyzed the regulation of ion channels (mechanoreceptors) following mFUS (AIP 15 W, 24 h after mFUS), focusing on piezo-type mechanosensitive ion channel component 1 (Piezo1), transient receptor potential canonical (Trpc) 1 and 6, transient receptor potential polycystic 2 (Trpp2), and transient receptor potential cation channel subfamily M member 4 (Trpm4), which are known to be affected by FUS-mediated shear stress, ultimately leading to changes in membrane permeability.^32^ Indeed, increased expression of mechanoreceptors was detected after mFUS, as demonstrated in both patient-derived, pure GSCs and more differentiated GBM cell cultures. Whereas GSCs responded to mFUS treatment with an increased expression of all investigated mechanoreceptors (focal area and peri-focally), more differentiated GBM cells were mainly characterized by an upregulation of Trpp2 and Trpm4 (focal area and peri-focally) (Figure 2E).

### Quantitative Polymerase Chain Reaction

To assess regional differences in gene expression profiles of GBOs or GBM cell cultures after mFUS treatment (AIP 11 or 15 W), the respective 3D hydrogel GBO/cell cultures were snap-frozen in liquid nitrogen and carefully removed from the Covaris tubes for sectioning. This procedure ensured that the orientation of the sample remained intact, allowing regional analysis of the mFUS-treated samples. Serial sections of the whole sample were prepared using a cryostat (#CM 1100, Leica Biosystems, Nussloch, Germany) at a consistent temperature of −20 °C to enable RNA isolation and immunofluorescence (see below) of sections from focal and peri-focal regions (center and margin). In the case of the GBOs, 100 sections each were pooled for the margin and the center, and in the case of the primary cells, 190 sections each were pooled for the margin and center and used for RNA processing, respectively.

RNA from the different samples was extracted using TRIzol® reagent (Invitrogen, Carlsbad, CA, USA) or the ARCTURUS® PicoPure® RNA isolation kit (Applied Biosystems, Foster City, CA, USA) according to the manufacturer’s instructions. DNase digestion, cDNA synthesis, reverse transcription, and qPCR were performed as described before^13,27–29^ using TaqMan primer probes (Applied Biosystems). Gene-specific primers and probes are listed in Supplementary Table 1. Cycles of threshold (*C*_T_) were determined, and the ∆*C*_T_ values of each sample were calculated as *C*_Tgene of interest_ − *C*_T glyceraldehyde-3-phosphate dehydrogenase (GAPDH)_. Samples with undetectable expression were excluded from mean expression calculations. The figures display either ∆*C*_T_ or linearized ∆*C*_T_ values (2 − ∆*C*_T_). The regulation of gene expression upon stimulation/treatment is shown as relative gene expression, calculated as *n*-fold expression changes = 2∆*C*_T_ control − ∆*C*_T_ stimulus.

### Immunofluorescence

For staining, cryostat sections were incubated with primary antibodies overnight at 4 °C, followed by incubation with secondary antibodies for 1 h at 37 °C and nuclei staining as described before.^13,28^ The embedded slides were analyzed by fluorescence microscopy (AxioObserver.Z1; Carl Zeiss AG, Oberkochen, Germany) using the ZEN 3.5 (blue edition) software (Carl Zeiss AG). The primary antibodies used are listed in Supplementary Table 2. If primary antibodies were derived from the same species, nonspecific binding was blocked with species-specific F_(ab)_ fragments (1:1000, from Jackson ImmunoResearch, West Grove, PA, USA). For negative controls, primary antibodies were omitted. Secondary antibodies were donkey anti-mouse or anti-rabbit IgG labeled with Alexa Fluor 488 or Alexa Fluor 555 (1:1000; Thermo Fisher Scientific). Double-positive cells were quantified manually by counting the number of cells positive for both markers, and assessing the absolute cell count in 2 to 7 GBOs for 2 independent GBO preparations for each region examined. Afterward, the mean values and standard deviations were calculated.

### Cytotoxicity Assay

After mFUS treatment, the cells were recovered from the 3D hydrogels using the Cell Recovery Solution (The Well Bioscience) according to the manufacturer’s protocol and subjected to further analysis. The cytotoxic effects were assessed using the CytoTox-Fluor™ Cytotoxicity Assay (Promega, Madison, WI, USA) according to the manufacturer’s instructions and as described.^11,13^ Supernatants from treated and control cells were collected at defined time points, and fluorescence was measured using a microplate reader (Infinite M200Pro, TECAN, Zürich, Switzerland) at 485/535 nm. Dead cell numbers were determined using a standard curve from digitonin-lysed cell dilutions (82.5 µg/ml; Merck Millipore, Darmstadt, Germany). The percentage of dead cells was calculated as the ratio of dead to total cells, as described before.^11,13^ Cell viability and proliferation were determined by counting viable cells using a hemocytometer at defined time points.

### Extreme Limiting Dilution Assay

The self-renewal capacity of primary cells pretreated with different conditions (control without mFUS, AIP 5 or 11 W, 48 h after mFUS) was assessed using an extreme limiting dilution analysis (ELDA), as described.^11,13^ The cell count was determined after mFUS and isolation of the cells from 3D hydrogels as described above, and serial dilutions (ranging from 1 to 3,200 cells per well) were plated in neurosphere medium. Cultures were maintained for 7 days, after which the number of spheres per well and the number of wells containing spheres (positive cultures) at each plating density were recorded. Data were analyzed using the ELDA online software tool (http://bioinf.wehi.edu.au/software/elda), and the results were plotted to estimate the self-renewal capacity. In addition, 5 × 10^4^ remaining cells/well were cultured in neurosphere medium for 7 days and processed for qPCR analysis.

### CD11b and CD3 Cell Depletion Using Magnetically Activated Cell Sorting

GBOs located more focally or peri-focally of the whole GBO-containing sample were picked from the 3D hydrogels immediately after mFUS treatment (AIP 11 W) and cultured separately for 24 h in GBO medium under gentle rotation. Subsequently, the regionally distinct GBOs were processed using MACS® technology to deplete CD11b- and CD3-expressing cells, including macrophages, microglia, T lymphocytes, and NK cells, as previously described.^27,33^ Specifically, single-cell suspensions were prepared from 400 mg of GBOs using the Neural Dissociation Kit (T) (Miltenyi Biotech GmbH, Gladbach, Germany), labeled with CD11b and CD3 MicroBeads (Miltenyi Biotech GmbH), and separated using MACS LS columns following the manufacturer’s protocol. Further analyses were performed by qPCR.

### Inhibition Experiments

Patient-derived more differentiated GBM cells were stimulated with the broad PI3-kinase inhibitor LY294002 (3 µM, #440204; Sigma−Aldrich; stock dissolved at 10 mM in DMSO). The inhibitor was administered 30 min (read out qPCR) to 1 h (read out Western blot) before mFUS (AIP 15 W) and during mFUS treatment using 3D hydrogel cell cultures, as described above. If a qPCR was subsequently performed, the samples were cultivated for a further 24 h without inhibitor stimulation; in the case of a Western blot, the samples were processed directly after mFUS.

### Western Blot

3D hydrogel GBO cultures or primary cell cultures from inhibiting experiments were prepared as described above. Twenty-four hours after the preparation of the 3D hydrogels, the samples were treated with mFUS (AIP 11 for GBOs, 15 W for LY294002-treated primary cells). Cells were recovered from hydrogels using the Cell Recovery Solution (The Well Bioscience) directly after mFUS in the case of LY294002-treated samples. In the case of GBOs, samples were cultured for an additional 48 h, followed by treatment with 50 µM Temozolomide (TMZ, stock dissolved at 100 mM DMSO; Sigma−Aldrich) for another 3 d. Afterwards, GBOs were picked from the 3D hydrogel. All samples were transferred into tubes containing lysis buffer (5 mM Tris−HCl (pH 7.8) supplemented with 10 mM NaCl, 0.1% (v/v) Triton X-100, 0.2 mM EDTA, 2 mM orthovanadate, and 1% (v/v) phosphatase inhibitor cocktail (100X, Thermo Fisher Scientific)) and homogenized using a tissue chopper (IKA-Ultra-Turrax®T 25 basic; IKA-Factory GmbH & Co. KG, Staufen, Germany). As described, 6-10 µg protein per sample was used for Western blotting experiments.^27^ Primary antibodies used were anti-MSI1 (1:500, mouse; MAB2628, R&D Systems, Minneapolis, MN, USA), anti-IGFBP5 (1:150, rabbit; sc-13093, Santa Cruz Biotechnology, Dallas, TX, USA), and anti-EphA5 (1:150, rabbit; sc-927, Santa Cruz Biotechnology), anti-phospho-GSK3β (Ser9) (1:250, rabbit; #9336, Cell Signaling, Danvers, MA, USA), and anti-phospho-Akt (Thr308) (1:200, rabbit; #9275, Cell Signaling) in 2% (w/v) casein/Tris-buffered saline with 0.1% tween (TBS-T) or 5% (w/v) bovine serum albumin/TBS-T for phosphorylated antibodies. The secondary antibody was donkey anti-rabbit IgG-HRP (1:12,500; A16035, Thermo Fisher Scientific) and donkey-anti-mouse IgG-HRP (1:10,000; A16011, Thermo Fisher Scientific) in 2% (w/v) casein/TBS-T. Equal protein loading was confirmed by stripping and incubating the membranes with anti-GAPDH (1:200; sc-47724; mouse, Santa Cruz) in 2% (w/v) casein/TBS-T with the secondary antibody donkey anti-mouse IgG-HRP (1:10000, A16011; Thermo Fisher Scientific) in 2% (w/v) casein/TBS-T as described.^27^

### Temozolomide Stimulation

3D hydrogel cultures of patient-derived differentiated GBM cells and GSCs were prepared as described above. Twenty-four hour after preparation, the 3D hydrogel cultures were treated with mFUS (AIP 5 and 11 W) and cultivated for 48 h. The cells were recovered from the 3D hydrogels using the Cell Recovery Solution (The Well Bioscience), seeded into 6-well plates at a density of 25,000 cells/well, and cultivated in the respective growth media supplemented with 50 µM TMZ (stock dissolved at 100 mM in DMSO; Merck Millipore) for up to 10 days. Controls were stimulated with an equal volume of DMSO (0.01% (v/v)) or cultivated in growth medium without any supplements (untreated control). Cytotoxic effects of TMZ and DMSO treatment following mFUS were investigated with the CytoTox-Fluor™ Cytotoxicity Assay (Promega) according to the manufacturer’s instructions and as described above.

### Statistical Analysis

The data were statistically analyzed using the GraphPad Prism 8.4® software (GraphPad Software, San Diego, CA, USA). Depending on the experimental setup, a Student’s *t*-test or a one-way or two-way ANOVA was performed, as indicated for each experiment in the figure captions. The sample size is stated in the figure captions. Statistical significance is marked with asterisks depending on the *P*-value: * *P* < 0.05, ** P < 0.01, and *** P < 0.001.

## Results

### mFUS Promotes Increased Expression of Dormancy- and Stemness-Associated Markers in GBOs in a Complex, Region- and Marker-Dependent Way

First, we analyzed the impact of mFUS (AIP 11 W, 24 h after mFUS) on patient-derived GBOs as complex, in vivo-adapted cellular systems cultured in 3D hydrogels. The characterization and quality control of the used GBOs are illustrated in the Materials and Methods section and shown in Figure 1A-C. The mFUS setup was validated as described in the Materials and Methods section and shown in Figure 2A-E. Markers studied in the following section were Sloan–Kettering Institute (SKI), insulin-like growth factor-binding protein 5 (IGFPB5), ephrin receptor A5 (EphA5), and histone cluster 1 H2B family member K (H2BK) as known dormancy-associated markers, and octamer binding transcription factor 4 (OCT4), Nestin, sex-determining region Y-box 2 (SOX2), and Musashi (Drosophila) homolog 1 (MSI1) as known stemness-associated markers.^11–15,28,29^ Notably, mFUS-treated sections referred to as margins were GBOs from peri-focal regions, whereas sections referred to as the center were in the focal region of mFUS. Sections referred to as controls obtained no mFUS-treatment.

**Figure 3.**
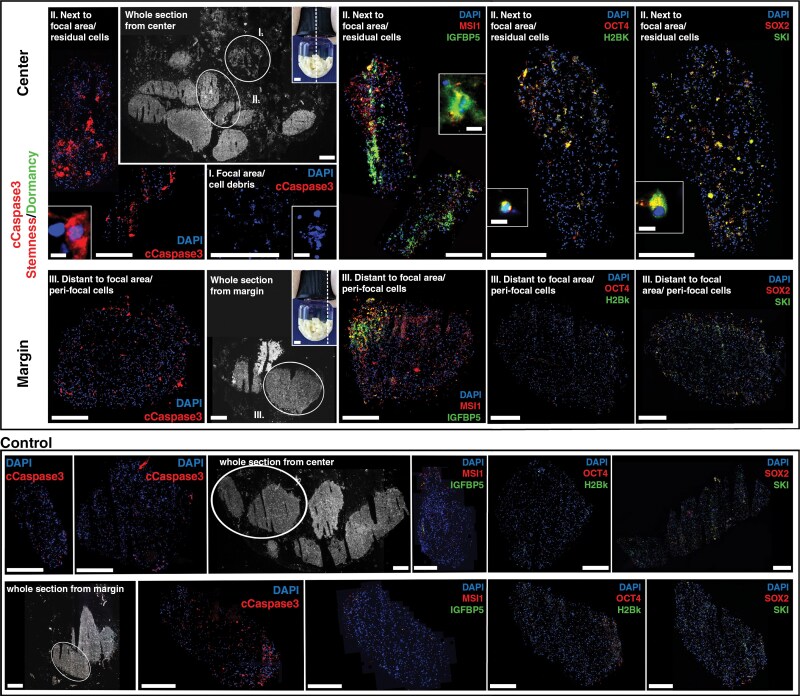
mFUS-treated (average incidence power 11 W) exemplary GBO-containing sample was cultured for another 24 h, cryofixed, and serially sectioned. Multi-immunofluorescence staining of sections from the sample’s focal (center) and peri-focal (margin) mFUS regions were performed with cCaspase3 (cell death marker), MSI1 (stemness marker), IGFBP5 (dormancy marker), OCT4 (stemness marker), H2BK (dormancy marker), SOX2 (stemness marker), and SKI (dormancy marker). Fragmented cell nuclei were observed in the focal region (I) after mFUS treatment, and cCaspase3 appeared predominantly near the focal region (II) compared with more distant regions (III). The untreated control showed a low incidence of cCaspase3. Prominent co-staining of MSI1 and IGFBP5 was found in both the sample´s center (residual cells) and the margin (peri-focal cells), while co-staining of OCT4 with H2BK and SOX2 with SKI showed increased expression compared to the untreated control, mainly in residual cells next to the focal area. Bar: 0.5 mm (whole sections), 1 mm (inserts with mFUS-tubes), 400 µm (GBO samples), 10 µm (inserts). Circled GBOs in whole sections show those GBOs that were visualized in detail in the immunofluorescence. H2BK, histone cluster 1 H2B family member K; IGFPB5, insulin-like growth factor-binding protein 5; SKI, SKI proto-oncogene. For further abbreviations, please refer to the previous figures.

Using immunofluorescence multi-staining, a region- and marker-dependent induction of dormancy- and stemness-associated molecules was measured depending on the GBO preparation examined, eg for the combinations MSI/IGFBP5, OCT4/H2BK, and SOX2/SKI (Figure 3). In the GBO specimen illustrated, the combination MSI/IGFPB5, in particular, was induced after mFUS, both in the residual cells next to the focal area and peri-focally. However, OCT4/H2BK and SOX2/SKI also showed increased protein expression compared to the controls without mFUS. Although these results differed between individual GBO preparations, quantification of double-positive cell populations after mFUS (2-7 GBOs of 2 independent GBO preparations) revealed an overall increase in the double-positive cells of the mFUS-treated samples (10.2 ± 4.57% and 8.0 ± 2.46% double-positive cells from the center/margin of mFUS-treated samples in case MSI/IGFPB5 [untreated controls with 1.0 ± 0.6% and 0.5 ± 0.4% double-positive cells], 16.6 ± 5.55% and 22.0 ± 12.84 % in case of OCT4/H2BK [controls 4.0 ± 2.6% and 2.4 ± 1.0%], and 15.5 ± 9.41% and 15.0 ± 9.39% in case SOX2/SKI [controls 3.0 ± 0.5 % and 9.9. ± 7.0%]). Analysis of cell death induced by mFUS revealed a significantly increased occurrence of cCaspase3-positive cells next to the focal area (particularly in areas that did not show clear expression of dormancy- and stemness-related molecules). In contrast, only a low abundance of cCaspase3-positive cells was observed in the peri-focal regions, comparable to the controls (Figure 3).

**Figure 4.**
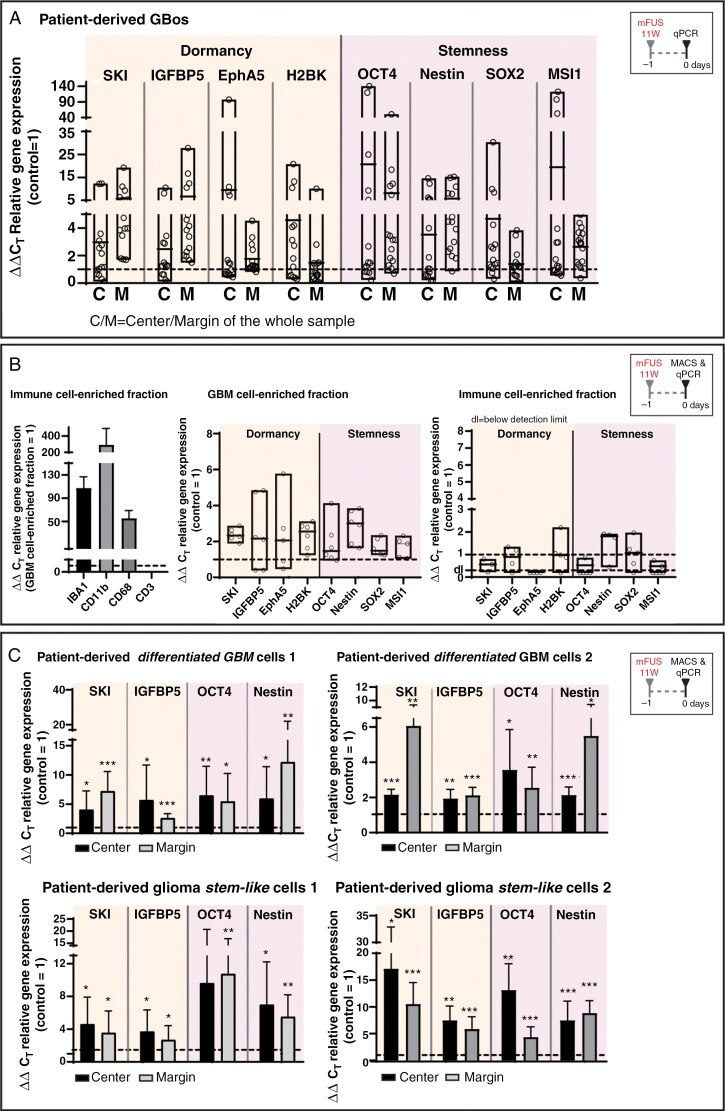
(A) GBOs treated with mFUS (average incident power 11 W, further cultured for 24 h) showed an induction of the mRNA expression of exemplary dormancy- and stemness-associated markers in the residual/peri-focal cells in a complex marker- and region-dependent manner (center/margin of whole samples) compared to average unstimulated controls (control = 1); *n* = 8 biological replicates with *n* = 1-2 technical replicates each. EphA5, ephrin receptor A5. (B) mFUS-treated (average incidence power 11 W) GBOs-containing samples were further cultured for another 24 h, and CD11b/CD3 MACS-based depletion of immune cells was performed. Left: Exemplary data on the success of MACS-based immune cell depletion is shown. Middle: The GBM cell-enriched fraction showed increased mRNA expression of dormancy- and stemness-associated markers. Right: The immune cell-enriched fraction showed significantly lower inductions or no detection of dormancy- and stemness-associated markers. Control = 1: average unstimulated controls; *n* = 3 biological replicates with *n* = 1-2 technical replicates each. (C) Pure cultures of patient-derived GSCs and more differentiated GBM cells were treated with mFUS (average incident power 15 W, 24 h after mFUS treatment; 2 different cell preparations each). Induction of the mRNA expression of exemplary dormancy (SKI, IGFBP5) and stemness markers (OCT4, Nestin) could be determined in residual/peri-focal cells in a complex marker- and region-dependent manner (center/margin of whole samples) compared to average unstimulated controls (control = 1); *n* = 3 biological replicates with *n* = 1-2 technical replicates each. Significant differences compared to the untreated control were determined by a nonpaired *t*-test and are indicated directly above the bars (* *P* < 0.05; ** *P* < 0.01; *** *P* < 0.001). Error bars correspond to the standard deviation. Please also refer to Supplementary Fig. 1 for expression of dormancy-/stemness-associated molecules with increasing mFUS power in GSCs. For abbreviations, please refer to the previous figures.

Accordingly, various GBOs showed an increased mRNA expression of dormancy- and stemness-associated molecules depending on the region studied and the markers analyzed (Figure 4A). A clear induction of EphA5, H2BK, OCT4, SOX2, and MSI1 was measured in most cases, especially near the focal region (center). In contrast, the mRNA expression of IGFBP5 and SKI was especially induced in peri-focal regions (margin) after mFUS treatment. Nestin showed a uniform induction both near the focal region and peri-focally (Figure 4A). However, due to the pronounced intra- and intercellular heterogeneity of GBOs, the various effects differed between the individual GBO preparations (no statistical significance could be obtained overall from preparations), but were clearly detectable on average (Figure 4A).

To obtain an initial indication of the cellular source of the increased expression of dormancy- and stemness-associated markers after mFUS, exemplary GBO preparations with/without mFUS treatment were subjected to tissue processing with subsequent depletion of CD11b- and CD3-positive immune cells using MACS. After validation of the successful depletion of the immune cells (Figure 4B, left), a determination of the mRNA expression of dormancy- and stemness-associated markers showed an increased expression of almost all markers to varying degrees (~2-fold and above on average) in the GBM cell-enriched fraction after mFUS (Figure 4B, middle), while only a slight increase of H2BK, Nestin, and SOX2 or no expression (EphA5) was observed in the immune cell-enriched fraction (Figure 4B, right). This finding indicated that the tumor cells themselves responded to mFUS treatment.

### mFUS-induced Expression of Dormancy- and Stemness-associated Markers Can be Attributed to Specific GBM Cell Subpopulations

To substantiate the findings of the GBOs and to clarify whether mFUS further promoted the stemness and dormancy properties of patient-derived GSCs or whether more differentiated patient-derived GBM cells developed an enhanced dormancy- and stemness-associated potential by mFUS treatment, 3D hydrogel-based, pure cultures of GSCs and more differentiated GBM cells were subjected to mFUS (AIP 15 W, 24 h after mFUS treatment) (Figure 4C).

Both pure cultures of GBM subpopulations (2 independent preparations of GSCs and more differentiated GBM cells each) responded to mFUS treatment with increased mRNA expression of dormancy- and stemness-associated markers, with the most consistent findings for SKI, IGFBP5, OCT4, and Nestin. The induction of expression was detectable both next to the focal region (center) and peri-focally (margin), ranging from ~2.5- to 16-fold induction compared to controls without mFUS, depending on the markers, samples, and regions examined (Figure 4C). Exemplified for GSCs, these effects were also detectable at lower mFUS settings (AIP 1, 5, and 11 W; Supplementary Fig. 1).

Thus, the mFUS treatment not only enhanced stemness- and dormancy-associated molecules in GSCs but also induced phenotypic changes in more differentiated GBM cells.

### mFUS Pretreatment Promotes Sphere Formation Ability and Chemoresistance in Different GBM Cell Subpopulations

**Figure 5.**
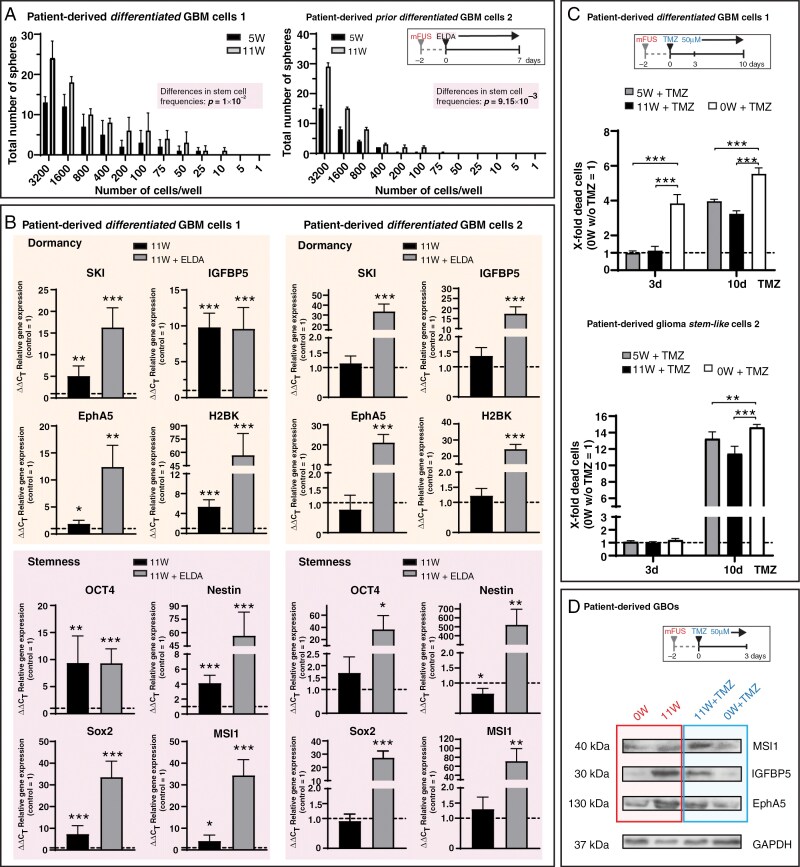
(A) mFUS (average incident power 5 and 11 W, 48 h after mFUS treatment) increased the ability of residual/peri-focal, formerly more differentiated GBM cells from patients to form cell-typical spheres when cultured under stem cell conditions as determined by the ELDA (2 different cell preparations with *n* = 2 biological replicates each). Briefly, cells were seeded at progressively lower densities, ranging from 3,200 cells per well to a single cell per well. Cultures were maintained until day 7 in neurosphere medium 48 h after mFUS treatment. At this point, the number of spheres per well and the number of wells containing spheres at each seeding density (number of positive cultures) were documented. Significance was tested using the online ELDA program 25 (https://bioinf.wehi.edu.au/software/elda/) with *P* = 0.01 for preparation 1 and *P* = 0.00915 for preparation 2. Refer to Supplementary Fig. 2 for the neurosphere formation assay of an example of an untreated control. (B) Determination of the mRNA expression of dormancy- and stemness-associated molecules 48 h after the mFUS treatment and continued cultivation for an additional 7 days in neurosphere medium compared to average unstimulated controls (control = 1). A separate analysis of the focal and peri-focal regions was not performed due to methodological constraints. Primary culture 1: *n* = 5 biological with *n* = 1-3 technical replicates each, primary culture 2: *n* = 4 biological with *n* = 1-3 technical replicates each. Significant differences compared to the untreated control were determined by a nonpaired *t*-test and are indicated directly above the bars (* *P* < 0.05; ** *P* < 0.01; *** *P* < 0.001). For abbreviations, please refer to the previous figures. (C) Pure cultures of patient-derived differentiated GBM cells and GSCs treated with mFUS (average incident power 5 and 11 W, 48 h after mFUS treatment stimulation with TMZ) showed higher resistance to TMZ (50 µM, treatment for 10 days), as evidenced by a lower number of dead cells compared to TMZ treatment alone (*n* = 2-3 biological replicates with *n* = 1-3 technical replicates each for differentiated GBM cells and GSCs, respectively; compared to average unstimulated controls (control = 1)). The significances between different stimulations were determined using a two-way ANOVA test followed by a Tukey’s multiple comparison test (** *P* < 0.01; *** *P* < 0.001). Error bars correspond to the standard deviation. (D) GBOs treated with mFUS (average incident power 11 W, 48 h after mFUS treatment, stimulation with 50 µM TMZ for 3 days) showed increased protein expression of MSI1 (stemness marker), IGFBP5 (dormancy marker), and EphA5 (dormancy marker) compared to untreated control. GAPDH served as a loading control. Refer to Supplementary Fig. 3 for full blots. For abbreviations, please refer to the previous figures.

To test the biological consequence of the increased expression of dormancy- and stemness-associated markers, pure preparations of more differentiated patient-derived primary GBM cells (2 independent preparations) cultured in 3D hydrogels were treated without or with mFUS (AIP 5 and 11 W), seeded in neurosphere medium after an additional 48 h, and further cultured for 7 days to analyze survivability and sphere formation under these conditions. The ability of residual/peri-focal, patient-derived, formerly differentiated GBM cells to form stem-like cell-typical spheres increased with intensified average incidence mFUS power (Figure 5A), even if the individual preparations responded to the mFUS treatment to varying degrees (example of an unstimulated control shown in Supplementary Fig. 2). Additionally, the observed increased ability to form spheres was supported by the mRNA expression profile of the GBM cells, exemplified for the GBM cells treated with AIP 11 W. Depending on the preparation, the mRNA expression of SKI, H2BK, Nestin, MSI1, and SOX2 (first GBM cell preparation), as well as OCT4, MSI1, and IGFBP5 (second preparation), was particularly well regulated in this experimental setting (no separate analysis of the focal and peri-focal region, as methodologically not possible). All these molecules showed induction by mFUS pretreatment, which was further supported by cultivation in a neurosphere medium (Figure 5B).

Since increased resistance to chemotherapy is also an essential hallmark of stem-like GBM cells, both pure cultures of patient-derived GSCs and more differentiated GBM cells (both with a methylated MGMT-promotor) were treated with 50 µM Temozolomide (TMZ; concentration nearly equivalent to that observed in human cerebrospinal fluid after oral administration^34^) 48 h after mFUS pretreatment (AIP 5 and 11 W) for a total of 10 additional days, and the cytotoxic effect of TMZ with or without mFUS pretreatment was tested. A significantly lower cytotoxic potential of TMZ (lower numbers of dead cells) was observed in the different GBM subpopulations pretreated with mFUS compared to GBM subpopulations without mFUS pretreatment (Figure 5C). However, in GSCs, this was not as pronounced as in the differentiated GBM subpopulation. Moreover, TMZ did not show a cytotoxic effect on the GSCs until after 10 days due to their stem cell nature. With increasing mFUS intensity, a slight increase in the protective effect of the mFUS pretreatment on the subsequent TMZ treatment was observed in both preparations examined, but this was not significant compared to the corresponding controls. Interestingly, GBOs treated with mFUS (AIP 11 W) and stimulated with TMZ for a further 3 days, 48 h after mFUS, also showed increased protein expression of MSI1, IGFBP5, and EphA5 compared to GBOs without mFUS pretreatment. Following the previous data, the sole effect of mFUS on the increased expression of the markers mentioned above was again observed here (Figure 5D; refer to Supplementary Fig. 3 for full blots).

### PI3-kinase Signaling Seems to be Involved in mFUS-mediated Phenotypic Changes

To gain a first insight into the signaling cascades involved in the mFUS-mediated effect of inducing the stemness and dormancy properties of residual/peri-focal GBM cells, we performed inhibition experiments in pure, more differentiated GBM cell cultures using the PI3-kinase inhibitor LY294002, followed by Western blot analysis of Akt (Thr308) and GSK3β (Ser9) phosphorylation, along with mRNA expression analysis of dormancy- and stemness-associated genes. Notably, the PI3-kinase/Akt pathway is frequently hyperactivated in GBM, supporting the maintenance and self-renewal of GSCs, contributing to tumor initiation and resistance to therapy.^35^ Further, previous studies, including our own, have further demonstrated that the PI3-kinase/Akt signaling pathway regulates the stemness and dormancy properties of GBM cells.^27,36^ Notably, the PI3-kinase (and Akt) activity is also known to be modulated by ROS.^37^

**Figure 6.**
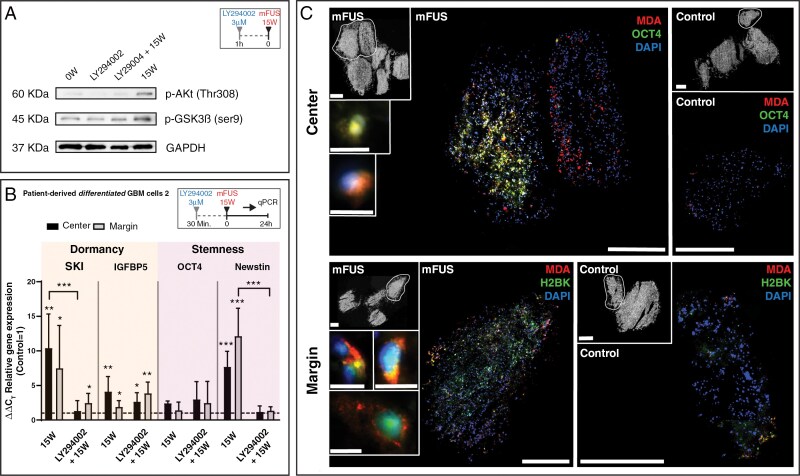
(A) Pure cultures of patient-derived differentiated GBM cells stimulated with the PI3-kinase inhibitor LY294002 (3 µM) and treated with mFUS (average incident power 15 W) showed decreased phosphorylation of Akt (Thr308) and GSK3ß (Ser9) compared to mFUS treatment alone in Western blot experiments. GAPDH served as a loading control. Refer to Supplementary Fig. 4 for full blots. (B) Pure cultures of patient-derived differentiated GBM cells stimulated with the PI3-kinase inhibitor LY294002 (3 µM) and treated with mFUS (average incident power 15 W) showed a partial reduction in the mRNA expression of dormancy- and stemness markers, evidenced by decreased expression of SKI (dormancy marker) and Nestin (stemness marker) compared to mFUS treatment alone; *n* = 2 biological replicates with *n* = 2 technical replicates each. The significance between different stimulations was determined using a one-way ANOVA test followed by a Tukey’s multiple comparison test (*** *P* < 0.001). Significant differences compared to the untreated control were determined by a nonpaired *t*-test and are indicated directly above the bars (* *P* < 0.05; ** *P* < 0.01; *** *P* < 0.001). Error bars represent standard deviation. (C) mFUS-treated (average incident power 11 W) GBO samples cultured for 24 h after mFUS and cryofixed for serial sectioning were stained with immunofluorescence. The staining was performed with MDA (an indirect indicator of ROS formation through lipid oxidation detection) co-stained with OCT4 (stemness marker) or H2BK (dormancy marker). MDA appeared predominantly in mFUS-treated samples compared to the untreated control. Additionally, prominent co-staining with MDA was observed in the center of the samples. Bar: 0.4 mm (whole sections), 500 µm (GBO samples), 10 µm (inserts). For abbreviations, please refer to the previous figures.

We demonstrated an effect of mFUS on the phosphorylation of Akt and GSK3β, which was partially prevented by LY294002 (Figure 6A; refer to Supplementary Fig. 4 for full blots). Importantly, mFUS-mediated Akt phosphorylation at Thr308 is an activating phosphorylation, while GSK3β phosphorylation at Ser9 inhibits GSK3β. Both ultimately lead to increased expression of molecules associated with dormancy and stemness (Figure 6B). Consequently, LY294002-mediated reduction of Akt and induction of GSK3β activation by inhibiting PI3-kinase resulted in a partial decrease in mFUS-induced expression of SKI and Nestin, especially, as dormancy- and stemness-associated molecules (Figure 6B). Thus, a complex intracellular process involving ROS activation, along with subsequent regulation by PI3-kinase/Akt/GSK3β, may caused changes in GBM cell phenotypes. Although these results offer only initial insights into the intracellular regulation of mFUS-mediated effects, especially concerning the involvement of mFUS-triggered ROS activation, they were further supported by clear co-staining of MDA (an indirect sign of ROS formation through lipid oxidation detection; see Figure 2) with OCT4 or H2BK, respectively, in mFUS-treated GBOs (Figure 6C).

In summary, ablation by mFUS seems to regulate the dormancy and stemness properties of the residual/peri-focally located GBM cells in a sophisticated region-specific manner.

## Discussion

FUS is a modern, noninvasive method used for diagnostic and therapeutic purposes.^1,38^ In addition to the more well-known FUS techniques,^19–21,39–43^ another mechanism for tissue ablation, namely mechanical high-intensity FUS (mFUS), is becoming increasingly important. High-intensity mFUS uses relatively short ultrasound bursts to minimize heating during ablation through acoustic cavitation.^8,39,41–43^ Indeed, mFUS-induced ablation has a good effect in treating various solid tumors,^4–6^ and phase I clinical trials have shown safety and efficacy in treating liver tumors, benign prostatic hyperplasia, and aortic valve calcification.^4–6^ Recent research showed mechanical ablation is a promising mechanism for treating malignant brain tumors.^4,5,7^ As progression of highly malignant GBMs is partially driven by the dormancy and stemness properties of surviving (residual/peri-focally located) GBM cells, a better understanding of the effect of mFUS on these modalities is certainly of particular importance. Thus, the present study investigated the effects of mFUS on stemness and dormancy properties of patient-derived GBOs and GBM primary cells as a novel therapeutic approach in an in vitro 3D hydrogel culture model.

We determined an increased expression of dormancy and stemness markers after mFUS in a complex region- and marker-dependent way, suggesting an effect of mFUS beyond the focal region. Accordingly, ELDA resulted in an increased ability of residual/peri-focal, formerly differentiated patient-derived GBM cells to form stem-like cell-typical spheres. This was accompanied by an increased expression of dormancy- and stemness-associated markers. Moreover, residual/peri-focal GBM cells were characterized by a higher resistance to TMZ, resulting in fewer dead cells than those treated solely with TMZ. At this, the mFUS-induced phenotypic changes appeared to be mediated by the PI3-kinase/Akt/GSK3β signaling pathway.

Dormancy describes a reversible cellular resting stage that plays a fundamental role in tumor evolution. Leaving this dormant state can lead to proliferation and tumor growth in primary tumor cells and metastases.^44^ In cellular dormancy, individual tumor cells can enter a cellular resting phase characterized by a reversible cell cycle arrest.^44^ Dormant and tumor stem-like cells share some similarities.^16,17^ Indeed, depending on the environmental stress (eg treatment), GBM cells can switch to a more dormant/stem cell-associated phenotype to survive unfavorable environmental conditions.^11,13^ We showed that residual/peri-focally localized GBM cells responded to mFUS treatment with increased dormant/stem-like cell properties. This observation regarding the influence of mFUS on dormancy/stemness properties beyond the focal region is novel and particularly important.

A probably peri-focal effect of FUS has already been demonstrated. For example, Qu et al.^45^ showed that cavitation-based focal ablation could stimulate local tumor infiltration by immune cells and promote inflammation at tumor sites that were not directly affected.^19,45^ This shows the reactivity of the peri-focal area after mFUS, which, in addition to the intensity of the sonication, is of decisive importance for the (long-term) consequences of mechanical ablation.

Regarding the influence of FUS on stem cells or stem-like cell properties, the most common previous findings refer to the so-called phenomenon of stem cell homing. Here, the mechanical effects of FUS increase the ability of stem cells to extravasate into the tissue,^46^ helping these cells to migrate more efficiently into damaged tissues. However, a few studies also refer to the direct influence of FUS on stem cell or stem-like cell properties. Seo et al.^47^ showed that endogenous neural stem cells were activated after low-intensity FUS-induced blood–brain barrier modulation. The expression of SOX2 and Nestin was significantly upregulated, and the stem cell activity was higher 1 week after low-intensity FUS. Further, FUS-mediated hyperthermia could sensitize GSCs to radiation.^19,48^ Hyperthermia also sensitizes GSCs to radiation therapy by downregulating the Akt signaling pathway, a key mediator of stemness and self-renewal.^19,49^ Song et al.^50^ observed that low-intensity pFUS attenuated GSC biomarkers’ expression, promoted GSCs escape from G0 quiescence, and significantly weakened the Wnt and Hh pathways. Accordingly, the sonication’s frequency and intensity appear decisive for the resulting biological effects.

In summary, we showed that mFUS exerts complex effects on residual GBM cells regarding their dormancy and stemness properties. Given that these properties may play a crucial role in the progression of GBMs, the observed effects of mFUS might be of great interest, especially concerning combined mFUS and chemotherapy concepts. Due to the complexity of the effects, further studies need to be conducted to develop efficient mFUS-based therapy options.

### Limitation of the Study

The lack of in vivo validation restricts the broader translational interpretation of the findings. However, given the limited applicability of current rodent in vivo mFUS systems and the debated relevance of rodent models in recapitulating human GBM heterogeneity, we chose to concentrate on complex patient-derived, human-based 3D models. Moreover, further inhibition experiments targeting various points within signaling cascades must be conducted for a more precise elucidation, especially regarding the role of ROS (and mechanoreceptors) as a relevant factor in mFUS-mediated phenotypic changes.

## Supplementary Material

vdaf184_suppl_Supplementary_Material

## Data Availability

All data on which the paper’s conclusions are based are available to readers in the manuscript.
