# Supplementary material for: Effect of focused ultrasound-induced mechanical ablation on stemness and dormancy properties of residual/peri-focally localized glioblastoma cells
Source: Neurooncol Adv. 2025 Aug 30;7(1):vdaf184. doi: 10.1093/noajnl/vdaf184 (PMC12449158; doi:10.1093/noajnl/vdaf184)
Supplement: vdaf184_suppl_Supplementary_Material [file vdaf184_suppl_supplementary_material.zip › Supplementary Table 1_mFUS and Dormancy_revised_clean.docx]

| **Human** |  |  |
| --- | --- | --- |
| *BGN* | *biglycan* | Hs 00156076_m1 |
| *CD3* | *cluster of differentiation 3* | Hs01062241_m1 |
| *CD11b* | *integrin alpha M (ITGAM)* | Hs00167304_m1 |
| *CD31* | *platelet endothelial cell adhesion molecule (Pecam 1)* | Hs00169777_m1 |
| *CD68* | *cluster of differentiation 68* | Hs00154355_m1 |
| *CD133* | *cluster of differentiation 133* | Hs00195682_m1 |
| *EphA5* | *ephrin receptor A5* | Hs00300724_m1 |
| *FN1* | *fibronectin 1* | Hs00277509_m1 |
| *GAPDH* | *glyceraldehyde-3-phosphate dehydrogenase* | Hs99999905_m1 |
| *GFAP* | *glial fibrillary acidic protein* | Hs00157674_m1 |
| *H2BK* | *histone cluster 1 H2B family member K* | Hs00955067_g1 |
| *IBA1* | *calcium-binding adapter molecule 1* | Hs00610419_g1 |
| *IGFBP5* | *insulin-like growth factor-binding protein 5* | Hs00174435_m1 |
| *KLF4* | *Krüppel-like factor 4* | Hs 00358836_m1 |
| *MSI1* | *Musashi (Drosophila) homolog 1* | Hs00159291_m1 |
| *Nestin* | *neuroepithelial stem cell protein* | Hs00707120_s1 |
| *OCT4* | *octamer binding transcription factor 4* | Hs00999632-g1 |
| *Piezo1* | *piezo-type mechanosensitive ion channel component 1* | Hs00207230_m1 |
| *S100b* | *S100 calcium-binding protein B* | Hs00902901_m1 |
| *SKI* | *SKI proto-oncogene* | Hs01057032_m1 |
| *SOX2* | *sex-determining region Y-box 2* | Hs00602736_s1 |
| *Trpc1* | *transient receptor potential canonical 1* | Hs00608195_m1 |
| *Trpc6* | *transient receptor potential canonical 6* | Hs00988479_m1 |
| *Trpm4* | *transient receptor potential cation channel subfamily M member 4* | Hs00214167_m1 |
| *Trpp2* | *transient receptor potential polycystic 2* | Hs00960946_m1 |
