# Supplementary material for: Effect of focused ultrasound-induced mechanical ablation on stemness and dormancy properties of residual/peri-focally localized glioblastoma cells
Source: Neurooncol Adv. 2025 Aug 30;7(1):vdaf184. doi: 10.1093/noajnl/vdaf184 (PMC12449158; doi:10.1093/noajnl/vdaf184)
Supplement: vdaf184_suppl_Supplementary_Material [file vdaf184_suppl_supplementary_material.zip › Supplementary Table 2_mFUS and Dormancy_revised_clean.docx]

| **Marker** | **Specificity** | **Epitope/dilution** | **Source** |
| --- | --- | --- | --- |
| cCaspase3 | *cleaved Caspase 3* | Polyclonal rabbit anti-human IgG; 1:200 | Cell Signaling Technology; Cat. #9661 |
| CD3 | *cluster of differentiation 3* | Polyclonal rabbit-anti-human IgG; 1:200 | Thermo Fisher Scientific; Waltham, MA, USA; Cat. PA5-29387 |
| CD11b | *integrin alpha M (ITGAM)* | Monoclonal mouse-anti-rat/human IgG; 1:250 | Santa Cruz Biotechnology; Cat. #sc-1186 |
| CD68 | *cluster of differentiation 68* | Monoclonal mouse-anti-human IgG; 1:200 | Santa Cruz Biotechnology; Cat. #sc-20060 |
| CD133 | *cluster of differentiation 133* | Polyclonal goat-anti-human IgG; 1:200 | Santa Cruz Biotechnology; Cat. #sc-23797 |
| GFAP | *glial fibrillary acidic protein* | Monoclonal mouse-anti-human IgG; 1:500 | Sigma-Aldrich, Taufkirchen, Ger; Cat. MAB3402 |
| H2BK | *histone cluster 1 H2B family member K* | Rabbit anti-human IgG; 1:400 | Biorbyt, Cambridge, UK; #orb184226 |
| Iba1 | *calcium-binding adapter molecule 1* | polyclonal goat-anti-human IgG; 1:1000 | Abcam, Cambridge, UK; Cat. #ab5076 |
| IGFBP5 | *insulin-like growth factor-binding protein 5* | Polyclonal rabbit anti-human IgG; 1:400 | Santa Cruz Biotechnology; Cat. #sc-13093 |
| Ki67 | *antigen Kiel 67* | Polyclonal rabbit-anti-human IgG; 1:200 | Santa Cruz Biotechnology; Cat. #sc-15402 |
| KLF4 | *Krüppel-like factor 4* | Monoclonal rabbit-anti-human IgG; 1:100 | Sigma-Aldrich; Cat. MAB-37846 |
| MDA | *malondialdehyde* | Monoclonal mouse-anti-human IgG; 1:50 | Thermo Fisher Scientific; Cat. #MA5-27559 |
| MSI1 | *Musashi (Drosophila) homolog 1* | Monoclonal mouse-anti-rat/human IgG; 1:200 | R&D Systems, Minneapolis, Canada; Cat. MAB2628 |
| OCT4 | *octamer binding transcription factor 4* | Polyclonal rabbit-anti-human IgG; 1:200 | Cell Signaling Technology, Danvers, USA; Cat. #2750 |
| S100b | *S100 calcium-binding protein B* | Monoclonal rabbit-anti-human IgG; 1:5000 | Sigma-Aldrich; Cat. S2644 |
| SKI | *SKI proto-oncogene* | Polyclonal rabbit anti-human IgG; 1:800 | Invitrogen Thermo Fisher Scientific; Cat. PA5-66852 |
| SOX2 | *sex-determining region Y-box 2* | Polyclonal rabbit-anti-human IgG; 1:200 | Santa Cruz Biotechnology, Dallas, Texas; Cat. #sc-20088 |
| vWF | *von Willebrand factor* | Monoclonal mouse-anti-human IgG; 1:100 | Santa Cruz Biotechnology; Cat. #sc-53465 |
