# Supplementary material for: Effect of focused ultrasound-induced mechanical ablation on stemness and dormancy properties of residual/peri-focally localized glioblastoma cells
Source: Neurooncol Adv. 2025 Aug 30;7(1):vdaf184. doi: 10.1093/noajnl/vdaf184 (PMC12449158; doi:10.1093/noajnl/vdaf184)
Supplement: vdaf184_suppl_Supplementary_Material [file vdaf184_suppl_supplementary_material.zip › Supplementary Table and Figure Legends.docx]

**Supplementary Table captions**

**Supplementary Table 1:** Identifiers of TaqMan assays with gene-specific primers and probes.

**Supplementary Table 2:** Antibodies used for immunohistochemistry.

**Supplementary Figure captions**

**Supplementary Figure 1:**  Pure culture of patient-derived glioma stem-like cells (GSCs) was exemplarily treated with different lower mechanical focused ultrasound (mFUS) settings (average incident power of 1W, 5W, 11W, read-out 24 h after mFUS treatment). Increasing mFUS power was found to partially induce the mRNA expression of exemplary dormancy marker Sloan-Kettering Institute (SKI), insulin-like growth factor-binding protein 5 (IGFBP5) and stemness marker octamer binding transcription factor 4 (OCT4) and Nestin, compared to average unstimulated controls (control =1); n= 3 biological replicates with n= 1-2 technical replicates each. Significant differences compared to the untreated control were determined by a non-paired *t*-test and are indicated directly above the bars (* *p* < 0.05; ** *p* < 0.01; *** *p* < 0.001). Error bars correspond to the standard deviation.

**Supplementary Figure 2:** Pure non-treated patient-derived prior differentiated GBM cells were used to perform an extreme limited dilution assay (ELDA). Briefly, cells were cultured under stem cell conditions and seeded at progressively lower densities, ranging from 3,200 cells per well to a single cell per well. Cultures were maintained until day 7 in neurosphere medium. At this point, the number of spheres per well and the number of wells containing spheres at each seeding density (number of positive cultures) were documented. Significances between published mechanical focused ultrasound (mFUS) stimulations were tested using the online ELDA program 25 (<https://bioinf.wehi.edu.au/software/elda/>).

**Supplementary Figure 3:** Full blots of glioblastoma organoids (GBOs) treated with mechanical focused ultrasound (mFUS) (average incident power 11 Watts, 48 h after mFUS treatment, stimulation with 50 µM Temozolomide (TMZ) for 3 days). Compared to the untreated control, increased protein expression was observed for Musashi (Drosophila) homolog 1 (MSI1; stemness marker), insulin-like growth factor-binding protein 5 (IGFBP5; dormancy marker), and ephrin receptor A5 (EphA5; dormancy marker). Glyceraldehyde-3-phosphate dehydrogenase (GAPDH) serves as a loading control. Refer to Fig. 5 for further information.

**Supplementary Figure 4:** Full blots of pure cultures of patient-derived differentiated GBM cells stimulated with PI3-kinase inhibitor LY294002 (3 µM, pretreated for 1 h prior mechanical focused ultrasound (mFUS)) and treated with mFUS (average incident power 15 Watts). Compared to mFUS treatment alone, LY294002 and mFUS stimulation showed decreased phosphorylation of Akt (Thr308) and GSK3ß (Ser9). Glyceraldehyde-3-phosphate dehydrogenase (GAPDH) served as a loading control. Refer to Fig. 6 for further information.
