# Supplementary figures and images for: Effect of focused ultrasound-induced mechanical ablation on stemness and dormancy properties of residual/peri-focally localized glioblastoma cells
Source: Neurooncol Adv. 2025 Aug 30;7(1):vdaf184. doi: 10.1093/noajnl/vdaf184 (PMC12449158; doi:10.1093/noajnl/vdaf184)

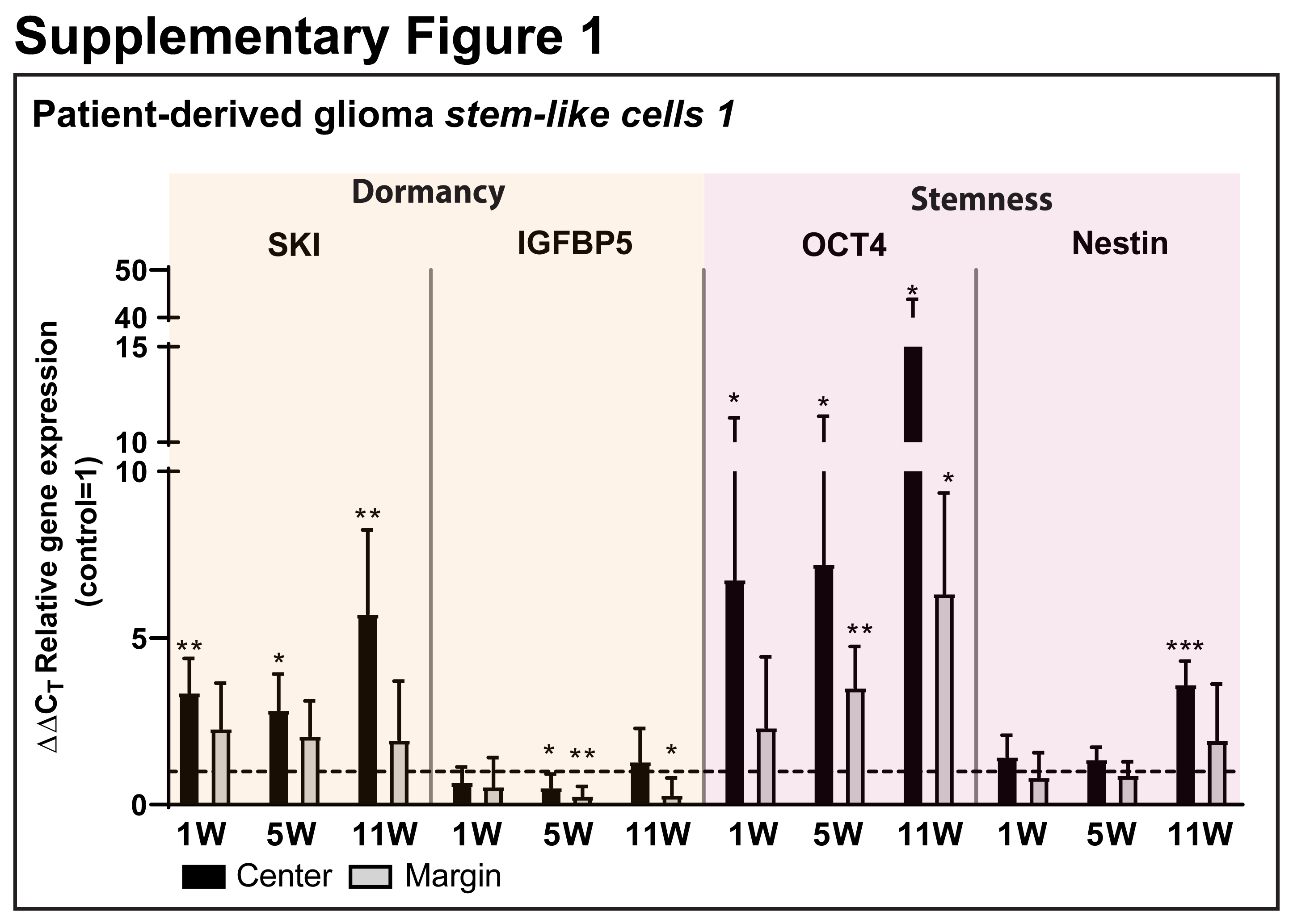

Supplement: vdaf184_suppl_Supplementary_Material [file vdaf184_suppl_supplementary_material.zip › Supplementary Figure 1_revised.tif]

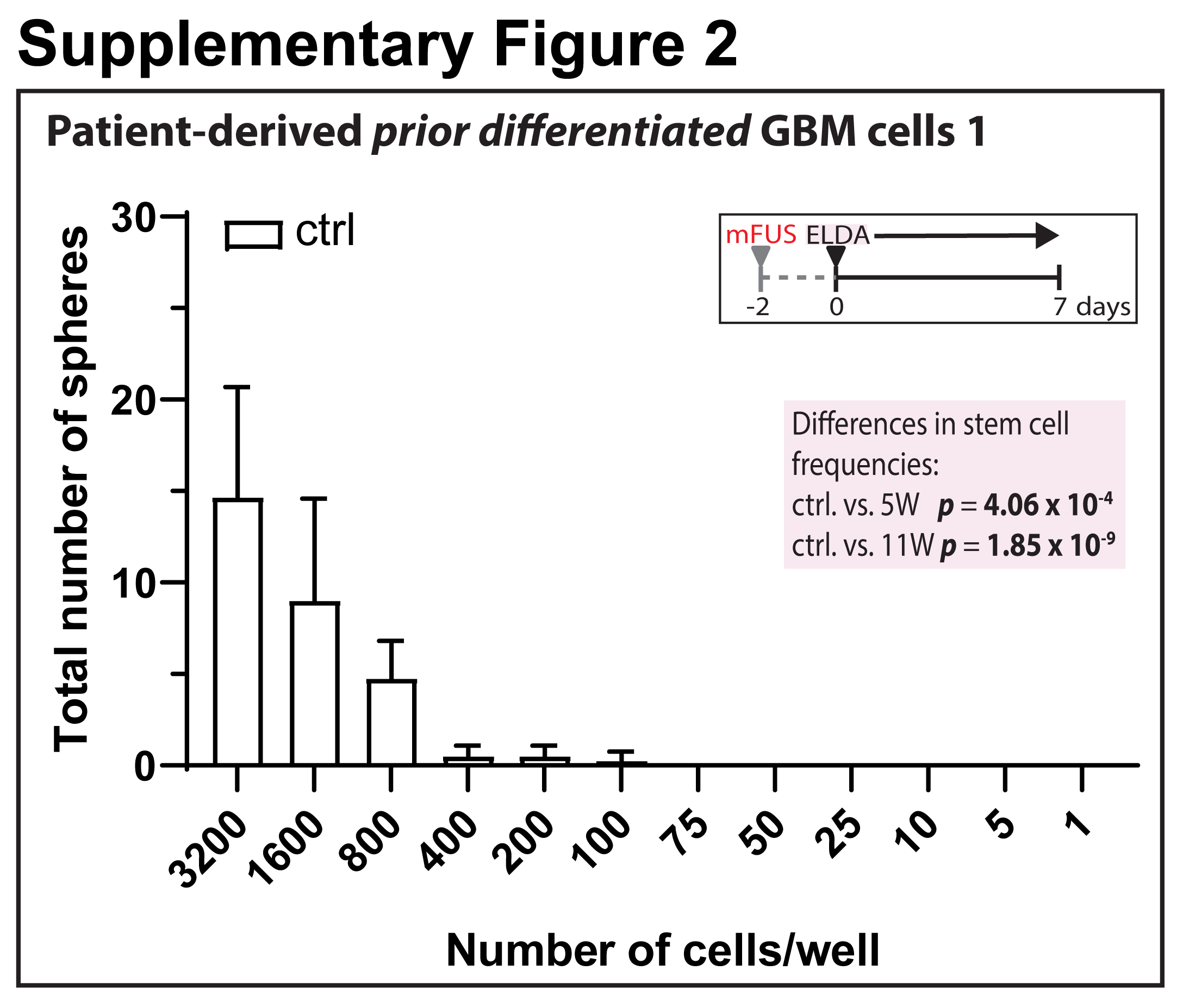

Supplement: vdaf184_suppl_Supplementary_Material [file vdaf184_suppl_supplementary_material.zip › Supplementary Figure 2_revised.tif]

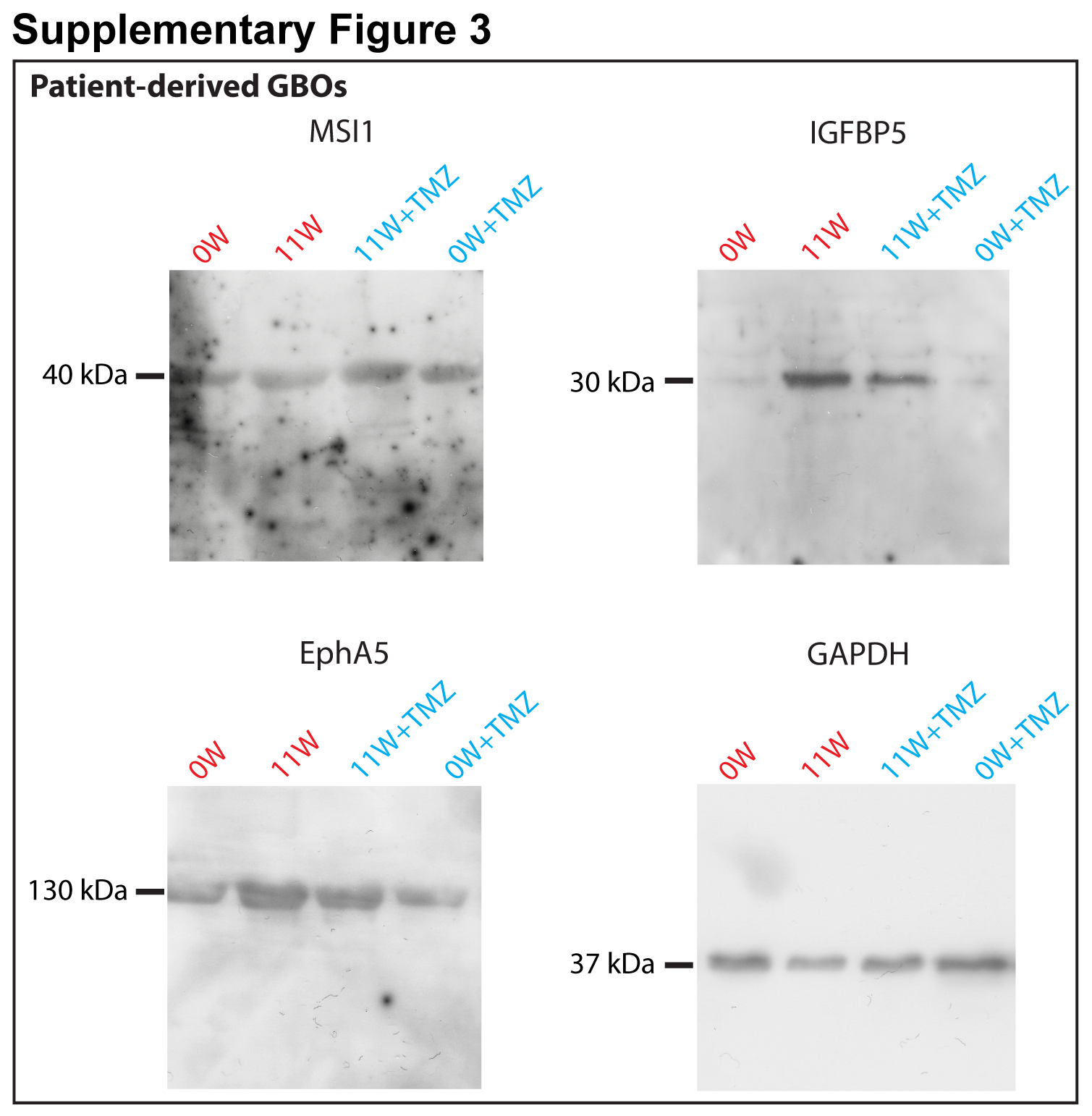

Supplement: vdaf184_suppl_Supplementary_Material [file vdaf184_suppl_supplementary_material.zip › Supplementary Figure 3.tif]

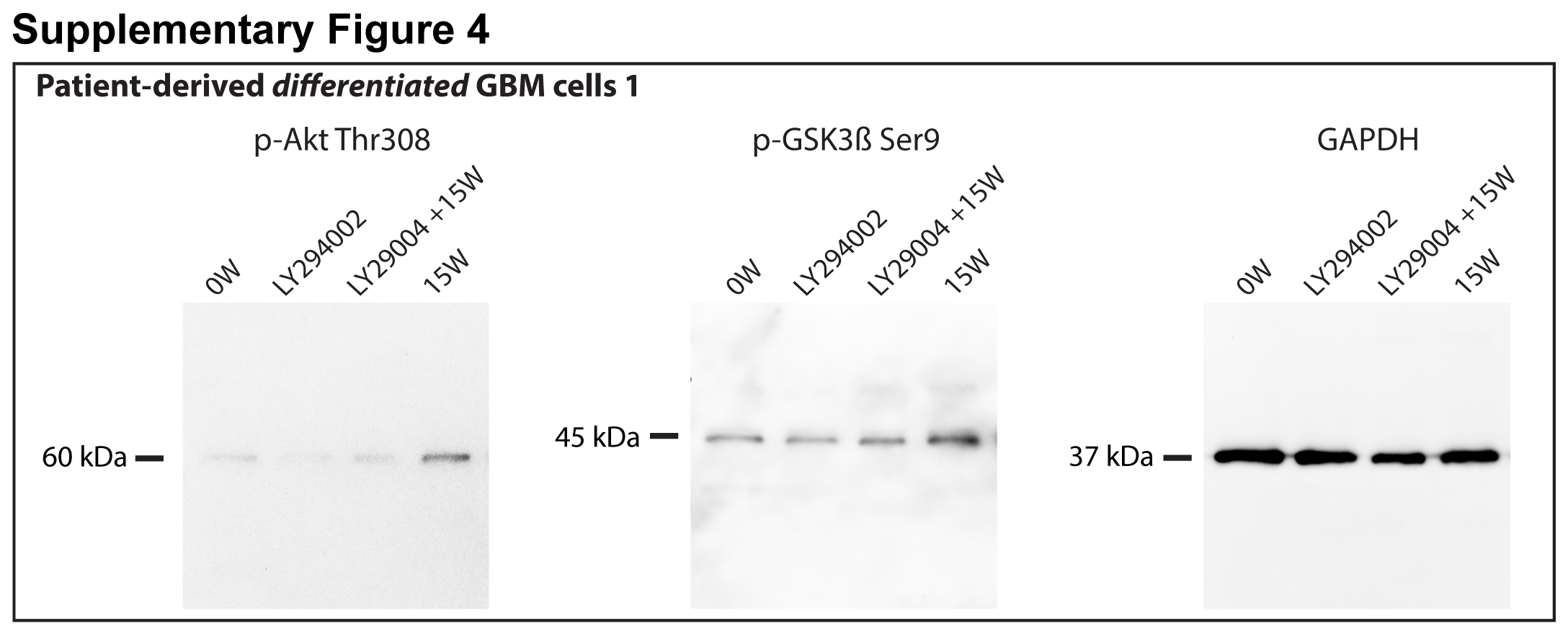

Supplement: vdaf184_suppl_Supplementary_Material [file vdaf184_suppl_supplementary_material.zip › Supplementary Figure 4_revised.tif]
